# Crime and punishment: Pakistan's legal failure to account for mental illness

**DOI:** 10.1192/bji.2020.30

**Published:** 2021-11

**Authors:** Romesa Qaiser Khan, Abdul Moiz Khan

**Affiliations:** 1 MBBS, King Edward Medical University, Lahore, Pakistan; 2 Second Year Internal Medicine Resident, Albany Medical Center, New York, USA. Email: abdulmoiz92@gmail.com

**Keywords:** Suicide, transcultural psychiatry, psychiatry and law, low and middle income countries, human rights

## Abstract

The Mental Health Ordinance 2001 was the last comprehensive legislation on mental health policy in Pakistan, replacing the Lunacy Act 1912. Since then, most of the amendments to the act have only delineated the jurisdiction of the provincial governments. Failure to account for mental illness in Pakistan brings with it unique challenges, such as the criminalisation of suicide and exploitation of blasphemy laws. There is a need for organised efforts to promote awareness of mental illness, amend the obsolete legislation in conformity with the scientific evidence, implement mental health policy effectively and deal with sensitive issues that have a strong sociocultural or religious background.

## The evolving crisis in Pakistani mental healthcare

The Pakistani healthcare system is no stranger to challenges. A dearth of infrastructure, constant cuts to a meagre health budget, overpopulated hospitals and overwhelmed physicians – by and large, the system in its entirety is either a marvel of medicine based on sheer human effort or a pariah exemplifying poor policy-making on the part of unconcerned politicians. Yet even in these less than ideal circumstances, there is a proverbial black sheep in the fields of medical care – mental illness is still a concept at best foreign to most and at worst challenged for its very existence.

To provide a brief insight, the World Health Organization's Mental Health Atlas Project gathers country-specific data in order to analyse progress towards achievement of objectives listed in the WHO Comprehensive Mental Health Action Plan for 2013–2020. According to the 2017 atlas,^1^ no mental health data had been compiled in a report for policy, planning or management purposes in the previous 2 years in Pakistan. The inadequacy of organised efforts at the level of institutions means that a lot of information cannot be reliably reported, but the few statistics we do have are alarming. The government's expenditure on mental health is 0.40% of the total health expenditure. Disability-adjusted life years due to mental illness work out at 2430.27/100 000 population. The suicide mortality rate is 2.9/100 000. There is neither a specific policy with implementable targets that can be monitored for progress, nor a dedicated authority to ensure its implementation.^1^ The 2009 WHO-AIMS report on Pakistan's mental health system estimated the numbers of psychiatrists and psychologists to be 342 and 478 respectively.^2^ Some more recent unofficial reports project the number of psychiatrists to be 500 serving a population of around 200 million. Given these figures, it is not surprising that mental health policy faces a crisis in practical implementation.

## History of mental health legislation in Pakistan

The Mental Health Ordinance 2001 was the last legislation passed for mental health policy, replacing the Lunacy Act 1912, which set out the provisions for admission, care and some legal rights of the ‘lunatic’.^3^ Under the ordinance, a mental health authority was to be established, comprising psychiatrists, psychologists and the Secretary of Health. This authority was not only to oversee the training and review of existing personnel and facilities, but also to formulate new policy for awareness and national standards of care. Much of the admission protocol from the Lunacy Act was retained and most of the guidelines for basic human rights of the mentally ill, including but not limited to their right to confidentiality, consent and appropriate treatment in case of attempted suicide. It also stipulated provisions for the examination of prisoners with mental diseases. Most of the amendments made to the act thereafter have only delineated the jurisdiction of the provincial governments and have not focused on any improvement in implementation or the adoption of a standard of care. No provincial act even exists for Baluchistan or Azad Jammu and Kashmir to date.^4^

## The legal framework and sample cases

Although ground rules of the approach to mental illness exist, the biggest challenge is the translation of these principles in the civil and criminal justice system. Civil courts have to deal with numerous cases related to ownership of assets/property, financial contracts and guardianship of people with mental illness.

In criminal law, chapter XXXIV, sections 464–475 of the Code of Criminal Procedures discuss the rights and exemptions for individuals without a ‘sound mind’. Ali & Saleem have described a series of cases in which the court had to adjudicate over an insanity plea from the accused. For example, there is a precedent of diagnosed mental illness such as schizophrenia leading to acquittal of the accused from charges of *Qatl-E-Amd* or intentional homicide. In another case, the absence of permanent infirmity in hypomania led to the dismissal of the accused's plea, on grounds of insanity, to postpone his trial for intentional homicide. In a third case, the accused entered an insanity plea after an initial confession of homicide. It was rejected on the grounds that there was no concrete evidence of mental illness.^5^

However, there are cases that have raised debates on fundamental human rights. In a controversial case, 50-year-old Imdad Ali was given the death penalty by a three-judge bench of the Supreme Court for murder of a cleric, despite confirmation of his paranoid schizophrenia by government psychiatrists who had treated him for the previous 8 years. The court stated that schizophrenia is ‘not a permanent mental disorder’, in direct contradiction to the ICD-10 and DSM-5 classifications of mental illnesses. The British Pakistani Psychiatrists Association (BPPA) wrote a letter to the Chief Justice of Pakistan bringing to light the inaccuracies of the procedure and requesting a panel of psychiatrists for an expert opinion.^6^ As of 2018, Imdad Ali was still on death row despite multiple appeals in his case, which were all rejected.

In another oversight, suicide and attempted suicide are deemed criminal offences under section 309 of the Pakistan Penal Code. The latter is punishable by incarceration of up to 1 year, or a fine or both. Owing to prevalence of Islamic beliefs that condemn suicidal behaviour and the fact that all attempted suicide cases must be registered by medico-legal centres (MLCs) before treatment, there is widespread hesitancy in disclosing such behaviour or seeking help for it. Pakistan provided no suicide mortality data to the WHO and did not count suicides among its annual mortality data until as recently as 2017.^7^ In February 2018, an amendment to the Mental Health Ordinance 2001 decriminalised attempted suicide and proposed the right to appropriate treatment, although the practical implementation remains to be seen.^8^

Perhaps the most sensitive application of mental health laws in Pakistani society comes in conjunction with the application of the blasphemy laws. The Pakistan Penal Code recognises a number of punishments for various degrees of the offence, the harshest one being death under section 295-C. In 2005, Saifullah Khan, who suffered from severe psychosis and delusions, was accused of blasphemy for alleged desecration of the Holy Quran. His appeal for bail was rejected by a Sessions Court despite the Standing Medical Board's confirmation that the accused was ‘unfit to plead’. The decision was later reversed by a High Court in 2006.^9^ In *Shahbaz Masih v State* in 2007 Masih was acquitted by Lahore High Court on account of having an ‘unsound mind’, but such success stories are few and far in between.^10^ Numerous times the victims have been extracted from police custody by mobs and beaten or burnt to death despite clear evidence of mental illness. In some cases, the blasphemy laws have even been used to frame or persecute minorities or individuals with disabilities such as Down's syndrome.^10^

## Conclusions and the next step

Mental illness is still subject to stigmatisation in Pakistani society. The lack of appreciation of the reality of mental diseases has grave impacts on the legal front as well. Not only is there is a dearth of infrastructure, but also the existing system is underutilised. Already difficult cases are fraught with the consequences of seeking help, either in the form of legal repercussions or social isolation. There is no denying that the ‘insanity plea’ is frequently used as a false defence. However, it is essential not to lose sight of people who are actually suffering from mental diseases that bar their capacity to have a ‘sound mind’ with a *mens rea* (criminal intent) and understanding of the consequences of their *actus reus* (criminal act). To that end, it is our recommendation that, in sensitive matters such as blasphemy cases, every mentally ill criminal should be reviewed by an independent psychiatric board to determine competency to stand trial, and law enforcement agencies should receive sensitivity training to tackle such cases in the field. Special courts should be set up for trial, and capital punishment should be excused for those who are proven to be mentally ill. Furthermore, an infrastructure for rehabilitation through therapy as an alternative to time in jail would serve a positive role in assimilating the mentally ill back into society. Prophylactically, a system of follow-up of individuals with a tendency to violence owing to mental illnesses or known history of harmful behaviour should be set up at the level of community hospitals and their attendants should be counselled on reducing risk. Lastly, public awareness is essential – we have to strive for a culture in which suicide attracts more compassion than infamy for the person and where the suffering of people battling mental illness is not invalidated at the level of the masses and the institutions.

## Supplementary material

For supplementary material accompanying this paper visit https://doi.org/10.1192/bji.2020.30. click here to view supplementary material
